# Motivations for Esports Betting and Skin Gambling and Their Association with Gambling Frequency, Problems, and Harm

**DOI:** 10.1007/s10899-022-10137-3

**Published:** 2022-07-08

**Authors:** Nancy Greer, Nerilee Hing, Matthew Rockloff, Matthew Browne, Daniel L. King

**Affiliations:** 1 Experimental Gambling Research Laboratory, School of Health, Medical and Applied Sciences, CQUniversity Australia, Melbourne, VIC Australia; 2 Experimental Gambling Research Laboratory, School of Health, Medical and Applied Sciences, CQUniversity, Bundaberg, QLD Australia; 3 College of Education, Psychology, and Social Work, Flinders University, Adelaide, Australia

**Keywords:** Esports betting, Skin gambling, Gambling motivations, Virtual items, Gambling problems, Gambling related-harm

## Abstract

This study aimed to examine gambling motivations for esports betting and skin gambling and their association with gambling frequency, problems, and harm. Data were collected via a cross-sectional online survey with 736 participants aged 18 + who engaged in esports cash betting (n = 567), esports skin betting (n = 180), or skin gambling on games of chance (n = 325). Respondents were asked to rate their motivations for the three activities across seven domains: social, financial, positive feelings or enhancement, internal regulation, skill building, competition/challenge, and skin acquisition. The results highlight both similarities and differences in gambling motivations across products. Financial gain and enhancement (i.e., excitement) were the main motivations endorsed for all activities, whereas skin acquisition was an additional motivation for esports skin betting and skin gambling. Across all three products, gambling to escape or improve mood was associated with higher levels of problem gambling and harm. Financial gain motivation was associated with problem gambling only for esports skin betting and skin gambling on games of chance. These findings underscore the importance of considering motivational influences on engagement with emerging gambling activities, especially since some motivations may be a contributing factor in harmful gambling outcomes.

## Introduction

Many new gambling products connected to video gaming have emerged in the last decade. Three popular products are esports cash betting, esports skin betting, and skin gambling on games of chance. *Esports cash betting* involves gambling money on video gaming competitions (esports), typically via wagering operators or dedicated esports betting providers. In contrast, *esports skin betting* involves using virtual video game items known as “skins” to bet on esports, most often via unregulated online operators (Greer et al., 2019). These unregulated online operators also often enable skin *gambling*, where skins are gambled on games of chance (i.e., roulette, jackpots). While participation in these emerging gambling activities is currently rare in general adult populations, participation amongst adolescents and young adults is proportionately much higher (Gambling Commission, 2019; Hing et al., 2021b; Hing et al., 2021; Russell et al., 2020). Early research also shows that esports betting and skin gambling are associated with higher levels of involvement in traditional gambling activities and gambling-related harm, especially for adolescents and young adults (Gainsbury et al., 2017b; Greer et al., 2021, 2022; Hing et al., 2021c, 2022; Wardle, 2019). However, participation in these emerging gambling activities only partially accounts for greater intensity of gambling involvement and harm, suggesting the influence of other underlying factors. One area of research which is important to explore is the motivations for gambling on these emerging products.

Several conceptual models identify key risk factors for gambling involvement and harm, including motivations for gambling (see Abbott et al., 2018; Binde, 2009). Motivation models identify key reasons why people gamble and how differences in gambling motivation type and strength are differentially associated with gambling participation and harmful gambling (i.e., Binde, 2013; Dechant, 2014; Lee et al., 2007; Stewart & Zack, 2008). To date little research exists on gambling motivations for esports cash betting, esports skin betting, or skin gambling on games of chance, and the role they play in contributing to gambling intensity and harm. To our knowledge, only one study has examined the impact of gambling motivations in relation to esports betting (Lelonek-Kuleta & Bartczuk, 2021). This study examined four gambling motivations amongst Polish esports bettors (enhancement, coping, social, and financial). However, insights from this study are limited because it measured motivations for all gambling activities combined, and not specifically for esports betting. It also defined esports betting as “esports or virtual sports betting” which conflates esports betting with betting on virtual sports. To our knowledge, no research has examined motivations for skin gambling. This paper addresses this knowledge gap by exploring seven potential gambling motivations for these three gambling products drawn from prior research and theorising: social, financial, positive feelings or enhancement, to regulate internal states, skill development, challenge or competition, and the acquisition of virtual items (skins).

### Social Motivations

Social reasons are important motives for gambling (Abarbanel, 2014; Dechant & Ellery, 2011; Flack & Morris, 2015; Francis et al., 2015; Lambe et al., 2015; Stewart & Zack, 2008; Wardle et al., 2011). The Gambling Motives Questionnaire (GMQ: Stewart & Jack, 2008), Reasons for Gambling Questionnaire (RGQ: Francis et al., 2015) and Lee et al’s (2007) five-factor gambling motivational measure all include similar social motivation items. These involve socialising with others, making a social gathering more enjoyable, and because friends are gambling. Social motivations have been linked with gambling on electronic gaming machines (EGMs) (Francis et al., 2015), sports, and card/casino table games (Abarbanel, 2014; Fang & Mowen, 2009; Flack & Stephens, 2019; Sundqvist et al., 2016). Similar social motivations may apply to esports cash betting, esports skin betting, and skin gambling given their similarity to these traditional gambling activities. Social interaction is also an important motivation for watching esports (Macey et al., 2020) and playing esports (Bányai et al., 2020; Lee & Schoenstedt, 2011; Weiss & Schiele, 2013), which could carry over into esports cash or skin betting. Furthermore, in line with research on buying virtual gaming items, gamblers may be engaged in esports skin betting or skin gambling to acquire skins to increase their social status amongst peers and in return receive more social interaction and friends (Calado et al., 2014; Cleghorn & Griffiths, 2015; Gainsbury et al., 2016; Hamari et al., 2017; Marder et al., 2019; Rockloff et al., 2020).

### Financial Motivations

Winning money is one of the strongest motivations for gambling reported by general populations of gamblers (Canale et al., 2015; Dechant, 2014; Flack & Morris, 2015; Francis et al., 2015; McGrath et al., 2010; Wardle et al., 2011), online gamblers (Abarbanel, 2014), and frequent gamblers (Lee et al., 2007). As with traditional gambling activities, esports cash betting is likely motivated by financial gain. In contrast, making a financial profit from esports skin betting and skin gambling requires that skins won are transferred out of the skin gambling website and sold for money through a skin exchange or by trading. The poor regulation of skin gambling and lack of consumer protection could result in players losing their skin inventories and the monetary value they have. Therefore, the added difficulty and risk of converting skins to money could result in esports skin bettors and skin gamblers being less motivated by financial gain than esports cash bettors.

### Enhancement Motivations

Gambling to experience positive feelings, often termed *enhancement*, include gambling for excitement, thrill, fun, entertainment, and enjoyment – as found in general populations of gamblers (Canale et al., 2015; Flack & Morris, 2015; Francis et al., 2015; Rockloff & Dyer, 2006; Wardle et al., 2011), online gamblers (Abarbanel, 2014), young adult gamblers (Lambe et al., 2015), probable pathological gamblers (Stewart & Zack, 2008), and frequent gamblers (Lee et al., 2007). Similar enhancement motivations have been found for online gaming (Demetrovics et al., 2011; Myrseth et al., 2017), spending money in virtual worlds (Mäntymäki & Salo, 2015), and buying other virtual items with chance-based contents known as “loot boxes” (Rockloff et al., 2020; Zendle et al., 2019). Similar to traditional gambling and video gaming activities, positive feelings such as excitement could be a key motivator for esports betting (cash or skins) and skin gambling. In addition, enjoyment of watching esports and/or playing video games could be enhanced by gambling on esports with skins.

### Regulation of Internal States (e.g., Escape, Improve Mood)

Gambling to regulate internal states is most often associated with escaping from or coping with negative emotions or thoughts, as well as relieving boredom or to relax (Abarbanel, 2014; Canale et al., 2015; Dechant, 2014; Flack & Morris, 2015; Francis et al., 2015; Lambe et al., 2015; Lee et al., 2007; Rockloff & Dyer, 2006; Stewart & Zack, 2008; Wardle et al., 2011). Escape is also an important motivation behind playing video and online games (Demetrovics et al., 2011; Frostling-Henningsson, 2009; Hilgard et al, 2013; Park, et al., 2011), watching esports (Hamari & Sjoblom, 2017), and playing esports (Weiss & Schiele, 2013). The motivation to escape for an individual could extend beyond general consumption of video games and esports into esports cash betting, esports skin betting, or skin gambling. Research has found that escape and coping motivations are particularly salient for gambling on chanced-based activities such as EGMs (Abarbanel, 2014; Fang & Mowen, 2009; Francis et al., 2015; Nower & Blaszczynski, 2010; Sundqvist et al., 2016; Thomas et al., 2009).

### Skill Building

Developing skills is a less common motivation for gambling and is mainly found for activities where knowledge and skill can be applied, such as sports betting (Gordon et al., 2015; Lamont &Hing, 2018) and poker (Hopley & Nicki, 2010). Esports betting, whether with money or skins, can also involve skills such as knowledge of esports players, teams, and the game. Furthermore, gambling with skins on esports and casino-style games could be a way to practice “real” monetary gambling or to improve gambling skills, as has been found in research on social casino games (Gainsbury et al., 2016).

### Competition and Challenge

The desire to compete with others and be challenged are also gambling motivations found in general gambling populations (Francis et al., 2015), online gamblers (Abarbanel, 2014), internet sports bettors (Lee et al., 2014), and at-risk gamblers (Sundqvist et al., 2016). Competition and challenge are also important motives for online gaming (Demetrovics et al., 2011; Park et al., 2011; Yee et al., 2012), buying loot boxes (Rockloff et al., 2020; Zendle et al., 2019), and playing esports (Bányai et al., 2020; Lee & Schoenstedt, 2011; Weiss & Schiele, 2013). For esports betting (cash or skins), motivations of competition and challenge may be embedded in the gambler’s perceived knowledge of esports games, players, and teams—which gives them both a (perceived) competitive edge against others and an intrinsically rewarding challenge. In contrast, skin gambling primarily involves simple versions of chance-based activities such as roulette, blackjack, jackpots, EGMs, coinflips, and case openings (Greer et al., 2020; Grove, 2016), which lack skilled play and arguably are less challenging for gamblers than esports betting. Additionally, the competition and challenge of gambling with skins on esports or games of chance may also relate to obtaining virtual items (skins), especially if they are unique.

### Acquisition of Virtual Items

Esports skin betting and skin gambling involve gambling with virtual items (skins). In addition to being able to exchange skins for money, skins won can be transferred from the gambling operator’s website to a video game player’s inventory, used in gameplay, or traded with other players (Greer et al., 2019; Grove, 2016). The motivation to gamble with skins to *obtain skins for gameplay* is supported by research on why gamers purchase virtual items in video games, social casino games, and loot boxes (Hamari et al., 2017; Hilgard et al., 2013; Hussain & Griffiths, 2014; Kim et al., 2016; Rockloff et al., 2020; Yee et al., 2012; Zendle et al., 2019). This research has found in-game purchases of virtual items, including loot boxes, are driven by motivations to customise the game and acquire in-game rewards or items. Another motivation for obtaining skins from gambling may be for *collectability*, particularly for rare or exclusive items (Cleghorn & Griffiths, 2015; Rockloff et al., 2020; Zendle et al., 2019). Amongst gamers aged 16–18 years (Zendle et al., 2019) nearly one-fifth (19.2%) were motivated to buy loot boxes “to gain specific items and characters, and to create a collection”. An Australian study found one of the top motivations for loot boxes purchasers was to “complete a set of items for a collection” (Rockloff et al., 2020). Lastly, another motivation for skin betting on esports or games of chance may be to *win skins to exchange for other skins*. Although not as prevalent as motivations to get rare or new items or for their collection, Rockloff et al. (2020) found around 40% of loot box purchasers were motivated to “have items to trade with others for more preferred items”.

### Gambling Motivations, Frequency, and Harm

Motivations for esports cash betting, esports skin betting, and skin gambling likely differ by product, which may have implications for differing outcomes for gambling frequency and gambling-related harm. Research on traditional forms of gambling has found that more frequent gamblers are more likely to endorse the gambling motives of *financial gain* (Flack & Morris, 2015; Francis et al., 2015; Rodrigeuz et al., 2015; Tabri et al., 2021), *enhancement* (Barrada et al., 2019; Francis et al., 2015; Lambe et al., 2015; Stewart & Zack, 2008), *coping* (Stewart & Zack, 2008), and *escape* (Flack & Morris, 2015; Thomas et al., 2009). Francis et al. (2015) also found regular gamblers to have higher scores than non-regular gamblers on motivations for *regulation of internal states, social,* and *challenge*. No research to date has explored how these gambling motivations may impact frequency of gambling on esports or with skins. The impact of motivations involving *skill building* and *acquisition of virtual items* on gambling intensity present a gap in the literature since they have not been explored for esports betting and skin gambling.

The primary gambling motivations associated with problem or harmful gambling are *enhancement* (i.e., excitement) (Browne et al., 2019; Flack & Morris, 2015; Francis et al., 2015; Lambe et al., 2015; Rockloff & Dyer, 2006; Rodrigeuz et al., 2015; Stewart & Zack, 2008; Wardle et al., 2011), *financial gain/money* (Browne et al., 2019; Lee et al., 2007; Russell et al., 2019; Tabri et al., 2021; Wardle et al., 2011), and *regulation of internal states* as measured by coping (Lambe et al., 2015; Stewart & Zack, 2008; Wardle et al., 2011) and escape (Browne et al., 2019; Flack & Morris, 2015; Rockloff & Dyer, 2006; Rodrigeuz et al., 2015; Thomas et al., 2009). The only study that has examined gambling motivations relating to the three emerging products explored here was in a sample of esports bettors using the GMQ-F measure (Dechant, 2014). It found that coping and financial motives were the strongest predictors of at-risk gambling (Lelonek-Kuleta & Bartczuk, 2021).

### Research Aims

Research into motivations for traditional gambling, video-gaming, and virtual item purchasing shows a considerable overlap between the types of motivations for engagement in these activities. Seven potential motivations for esports betting and skin gambling have been identified: social, financial, positive feelings or enhancement, regulation of internal states, skill building, competition/challenge, and the acquisition of virtual items (skins). This research aims to address the gaps in knowledge on esports betting and skin gambling, specially addressing two research questions:

1. What are the main gambling motivations for esports cash betting, esports skin betting, and skin gambling on games of chance, and do they differ for these three products?
2. Which gambling motivations for esports cash betting, esports skin betting, and skin gambling are associated with greater gambling frequency, problem gambling, and harm?

## Methods

### Participants and Procedure

The sample was recruited between October 2018-February 2019 via online crowdsourcing: (1) Amazon's Mechanical Turk, and (2) social media posts (Facebook, Twitter, Reddit) targeted to online video-gaming, esports, and gambling communities. Compensation for participants varied by source: participants recruited through Mechanical Turk received US$1.80 and social media participants entered a prize draw to win one of five $50 USD Amazon Gift Cards, or 1 × Samsung Galaxy Tablet. A total of 2952 respondents started the survey (Mechanical Turk = 1,949; social media posts = 1003). Of those, 766 were excluded because they did not meet the inclusion criteria of participating in esports cash betting, esports skin betting, or skin gambling in the last 6 months. A further 245 were excluded as they did not reside in one of four selected in-scope countries (USA, UK, Canada, Ireland). Another 248 were excluded for failing an attention check question, 34 excluded for being aged under 18 years, and 15 did not give consent for participation. Of the 1644 remaining respondents, 642 started but did not complete the survey, 213 were found to be duplicate responses, and 52 had poor data quality. A cull left a total of 737 completes, yielding a 25.0% response rate from eligible participants. One participant did not complete the gambling motivations questions.

This paper analysed the data for 736 participants who answered gambling motivation questions for one or more of the following activities they had *participated in during the last 6 months*: (1) esports cash betting (n = 576, 77.0%), (2) esports skin betting (n = 180, 24.5%), and (3) skin gambling on games of chance (n = 325, 44.2%). Table 1 provides demographic characteristics and gambling frequency for the final sample. The sub-sample was mostly male (80.2%) with an average age of 28.98 years (SD = 8.07). The majority resided in the USA (73.0%), were recruited via Mechanical Turk (79.9% vs 20.1% via social media), were single/never married (60.5%), had a university level education (59.1%), were employed (85.6%), and earned a low-to-medium annual income. A higher percentage of participants engaged in esports cash betting at least monthly (51.4%) compared to esports skin betting (15.2%) and skin gambling on games of chance (27.0%).

**Table 1:** Demographic characteristics and gambling frequency of the final sample (N = 736)

Variable	n = 736 (n, %)
*Age (scale)*	Mean = 28.98 years
	(*SD* = 8.07)
	Range: 18–64 years
*Gender*	
Male	590 (80.2)
Female	146 (19.8)
*Country of residence*	
USA	537 (73.0)
UK	119 (16.2)
Canada	71 (9.6)
Ireland	9 (1.2)
*Recruitment source*	
Mechanical Turk	588 (79.9)
Social media	148 (20.1)
*Marital status*	
Single, never married	445 (60.5)
Married/domestic partnership	269 (36.5)
Divorced/separated/widowed	22 (3.0)
*Highest level of education*	
Primary school	39 (5.3)
Secondary school	115 (15.6)
Post-secondary/tertiary	147 (20.0)
Bachelor/master/doctoral	435 (59.1)
*Employment status*	
Employed	630 (85.6)
Unemployed	106 (14.4)
*Annual personal income*	
$0—$19,999 per year	182 (24.7)
$20,000—$39,999 per year	175 (23.8)
$40,000—$74,999 per year	242 (32.9)
$75,000—$149,999 per year	93 (12.6)
$150,000 or more per year	13 (1.8)
Prefer not to say	31 (4.2)
*Esports cash betting frequency (last 6 months)*	
At least monthly	378 (51.4)
Less than monthly	209 (28.4)
Never	149 (20.2)
*Esports skin betting frequency (last 6 months)*	
At least monthly	112 (15.2)
Less than monthly	111 (15.1)
Never	513 (69.7)
*Skin gambling frequency (last 6 months)*	
At least monthly	199 (27.0)
Less than monthly	163 (22.1)
Never	374 (50.8)

## Measures

### Socio-demographics

Socio-demographic variables included age, gender, country of residence, marital status, education level, employment status, and annual personal income (see Table 1).

### Gambling Activities

Frequency of gambling participation was collected for esports cash betting, esports skin betting, and skin gambling on games of chance in the last 6 months: never (0), not in the last 6 months (1), at least 6 monthly (2), at least monthly (3), at least fortnightly (4), and at least weekly (5). For analyses, gambling frequency was recoded to 0 = less than monthly (codes 1–2), and 1 = at least monthly (codes 3–5). Table 1 shows gambling frequency of each product.

### Problem Gambling and Gambling-Related Harm

Problem gambling was measured using the Problem Gambling Severity Index (PGSI: Ferris & Wynne, 2001) for the last 6-month timeframe. The PGSI consists of 9-items rated on a 4-point rating scale: ‘never’ (0), ‘sometimes’ (1), ‘most of the time’ (2), and ‘almost always’ (3). Total scores range from 0 to 27 categorising gamblers by score into: non-problem (0), low-risk (1–2), moderate-risk (3–7), and problem gambling (8–27). The 10-item Short Gambling Harm Screen (SGHS, Browne et al., 2017) was used to measure gambling-related harm experienced from all gambling, over the last 6 months. Participants answered to experiencing each gambling-related harm (0 = no, 1 = yes), with total scores ranging from 0 to 10 and categorised into groups: 0 harms, 1–2 harms, 3–4 harms, and 5–10 harms. Table 2 shows descriptive statistics for PGSI and SGHS by sample group.

**Table 2:** PGSI and SGHS categorisation (n, % n) and mean scores by sample group (n = 736)

Variable	Esports cash bettor (n = 567)	Esports skin bettor (n = 180)	Skin gambler (n = 325)	Total sample (N = 736)
*Problem Gambling Severity Index (PGSI)*				
0 Non-problem, n (%)	112 (19.8)	32 (17.8)	42 (12.9)	129 (17.5)
1–2 Low risk, n (%)	119 (21.0)	43 (23.9)	75 (23.1)	164 (22.3)
3–7 Moderate risk, n (%)	170 (30.0)	50 (27.8)	98 (30.2)	219 (29.8)
8 + Problem, n (%)	166 (29.3)	55 (30.6)	110 (33.8)	224 (30.4)
Total, n (%)	567 (100.0)	180 (100.0)	325 (100.0)	736 (100.0)
Mean PGSI score (SD)	5.80 (6.23)	5.88 (6.27)	6.25 (5.94)	5.90 (6.16)
*Short Gambling Harm Screen (SGHS)*				
0 harms, n (%)	189 (33.3)	59 (32.8)	96 (29.5)	240 (32.6)
1–2 harms, n (%)	131 (23.1)	37 (20.6)	82 (25.2)	174 (23.6)
3–4 harms, n (%)	68 (12.0)	29 (16.1)	49 (15.1)	97 (13.2)
5–10 harms, n (%)	179 (31.6)	55 (30.6)	98 (30.2)	225 (30.6)
Total, n (%)	567 (100.0)	180 (100.0)	325 (100.0)	736 (100.0)
Mean SGHS score (SD)	3.02 (3.14)	2.99 (2.97)	3.05 (3.04)	2.98 (3.09)

### Gambling Motivations

The development and design of the gambling motivation items for esports cash betting, esports skin betting, and skin gambling was informed by: (1) a review of the literature of gambling, video gaming, and esports motivations, (2) validated gambling and gaming motivation questionnaires, and (3) data from 30 qualitative interviews conducted in March–June 2018 by the first author with regular esports bettors (cash or skins) and skin gamblers on games of chance (Greer, 2018). Table 3 summaries the source/s of each motivation item, noting in some instances wording was slightly modified.

**Table 3:** Sources for motivational items for esports cash betting (ECB), esports skin betting (ESB), and skin gambling (SG)

Motivation item	Motivation type	Product/s	Item source/s
To socialise	SOC	ECB, ESB, SG	GMQ; RGQ; FFGM; EGMQ
Because friends are gambling on it	SOC	ECB, ESB, SG	GMQ; RGQ; FFGM; EGMQ
To win money	FIN	ECB, ESB, SG	GMQ-F; RGQ; FFGM; Abarbanel (2014)
To win skins to exchange to money	FIN	ESB, SG	Qualitative interviews (NG)
For the excitement	ENH	ECB, ESB, SG	GMQ; RGQ; EGMQ
It enhances my enjoyment viewing the esports match	ENH	ECB, ESB	Qualitative interviews (NG)
It enhances my enjoyment viewing video games	ENH	SG	Qualitative interviews (NG)
To improve my gambling skills	SKILL	ECB, ESB, SG	MOGQ; Gainsbury et al (2016); Neighbors et al. (2002)
To learn how to gamble on similar types of gambling activities	SKILL	ECB, ESB, SG	Hollingshead et al. (2016)
To practice “real” money gambling	SKILL	ECB, ESB, SG	Hollingshead et al. (2016)
To escape from my worries	REG	ECB, ESB, SG	GMQ; RGQ; FFGM; MOGQ; EGMQ
To feel better/cheer up	REG	ECB, ESB, SG	GMQ; RGQ; EGMQ
To compete with others	CC	ECB, ESB, SG	RGQ; FFGM; MOGQ; Abarbanel (2014)
For the challenge	CC	ECB, ESB, SG	RGQ; Abarbanel (2014); Neighbors et al. (2002)
To win skins for my collection	SKIN	ESB, SG	Qualitative interviews (NG)
To win skins to exchange for other skins	SKIN	ESB, SG	Qualitative interviews (NG)
To win skins to use in video game play	SKIN	ESB, SG	Qualitative interviews (NG)

SOC = Social; FIN = Financial; ENH = Enhancement; SKILL = Skill building; REG = Regulation of internal states; CC = Competition /challenge; SKIN = Skin acquisition; GMQ = Gambling Motives Questionnaire (Stewart & Zack, 2008); RGQ = Reasons for Gambling Questionnaire (Wardle et al., 2011); GMQ = Gambling Motives Questionnaire-Financial (Dechant, 2013); FFGM = Five-Factor Gambling Motives Scale (Lee et al., 2007); MOGQ = Motives for Online Gaming Questionnaire (Demetrovics et al., 2011); EGMQ = Electronic Gaming Motives Questionnaire (Myrseth et al., 2017)

Participants were asked to rate their agreement with the gambling motivation items on a 5-point scale from 1 = strongly disagree to 5 = strongly agree for each of the three gambling activities they had gambled on in the last 6 months. A total of 18 items were asked, two items each for the social, enhancement, skill building, regulation of internal states (e.g., escape), and competition/challenge domains. An extra financial item, *“To win skins to exchange to money”*, was asked for esports skin betting and skin gambling, as were three items for skin acquisition. Table 4 shows the mean scores and total percentage agreed (score 4 or 5) for the gambling motivation items by gambling activity.

**Table 4:** Agreement for gambling motivations by gambling activity (mean score, SD; % agree/strongly agree)

Gambling motivation	Esports cash betting (n = 567)		Esports skin betting (n = 180)		Skin gambling (n = 325)	
	Mean (SD)	% Agree	Mean (SD)	% Agree	Mean (SD)	% Agree
*Social*						
To socialise	2.54 (1.33)	28.0	2.67 (1.39)	35.0	2.50 (1.30)	25.5
Because friends are gambling on it	2.70 (1.38)	32.6	2.87 (1.44)	40.0	2.69 (1.38)	32.9
*Financial*						
To win money	4.36 (0.91)	86.6	3.63 (1.36)	61.7	3.83 (1.26)	65.8
To win skins to exchange to money	–	–	3.81 (1.26)	63.9	3.82 (1.22)	68.0
*Enhancement*						
For the excitement	4.11 (0.96)	80.1	4.08 (1.02)	78.9	4.01 (0.98)	74.8
It enhances my enjoyment viewing the esports match	3.89 (1.09)	71.8	3.81 (1.21)	67.8	–	–
It enhances my enjoyment viewing video games	–	–	–	–	3.10 (1.28)	42.8
*Skill building*						
To improve my gambling skills	2.65 (1.36)	30.0	2.36 (1.33)	23.9	2.43 (1.36)	26.5
To learn how to gamble on similar types of gambling activities	2.60 (1.39)	29.8	–	–	–	–
To practice “real” money gambling	–	–	2.35 (1.38)	25.0	2.49 (1.40)	27.4
*Regulation of internal states*						
To escape from my worries	2.43 (1.39)	25.0	2.49 (1.42)	29.4	2.56 (1.41)	30.8
To feel better/cheer up	2.76 (1.38)	34.0	2.84 (1.38)	39.4	2.76 (1.35)	32.9
*Competition/challenge*						
To compete with others	3.21 (1.35)	51.9	3.32 (1.38)	55.0	3.17 (1.34)	47.7
For the challenge	3.58 (1.21)	62.8	3.51 (1.35)	58.3	3.33 (1.33)	53.2
*Skin acquisition*						
To win skins for my collection	–	–	3.99 (1.14)	74.4	3.93 (1.12)	72.0
To win skins to exchange for other skins	–	–	3.82 (1.19)	71.7	3.84 (1.09)	68.3
To win skins to use in video game play	–	–	3.93 (1.25)	72.2	3.90 (1.17)	71.7

*All motivation items rated on a scale from 1 = strongly disagree to 5 = strongly agree

### Data Analysis

Motivation items were averaged for composite scores and labelled as *social*, *financial*, *enhancement, skill building*, *regulation of internal states*, *competition/challenge*, and *skin acquisition* based on logical grouping of the items (Table 4). Internal consistency was assessed for each motivation domain using Cronbach’s alphas for esports cash betting, esports skin betting, and skin gambling independently. Cronbach’s alpha for social, financial, regulation of internal states and competition/challenge was based on only 2 items and therefore was equivalent to the between-item correlation. The *financial* motivation was a single item *“to win money”* for esports cash betting, and two items for esports skin betting and skin gambling with the addition of *“to win skins to exchange to money”*. After this adjustment, the gambling motivations domains with two items had good internal consistency for esports cash betting (from α = 0.61 to α = 84), esports skin betting (from α = 0.63 to α = 84), skin gambling (from α = 0.64 to α = 89). The exception was the *enhancement* motivation for skin gambling with a Cronbach’s alpha = 0.45. Given that the *“for the excitement”* item was a more important motivation for skin gambling (M = 4.01) than *“it enhances my enjoyment viewing video games”* (M = 3.10), only the former excitement item was retained for data analysis.

Independent t-tests, correlational, and descriptive analyses were conducted to explore differences in gambling motivations by age (years), gender (male, female), and gambling activity (esports cash betting, esports skin betting, and skin gambling). Significance testing on the differences in individual motivation items between esports cash betting, esports skin betting, and skin gambling could not be conducted given the samples across the three activities were not independent. Three univariate logistic regressions were conducted to examine, when controlling for age and gender, the independent contribution of the gambling motivations as predictors (IVs) of regular gambling (less than monthly/at least monthly) for esports cash betting, esports skin betting, and skin gambling (DVs). For each of the three gambling products, two ordinal regressions were conducted with age, gender, and gambling motivations as IVs and problem gambling severity (4 categories) and gambling-related harm (4 categories) as DVs.

## Results

### Gambling Motivations by Product

**Fig. 1:**
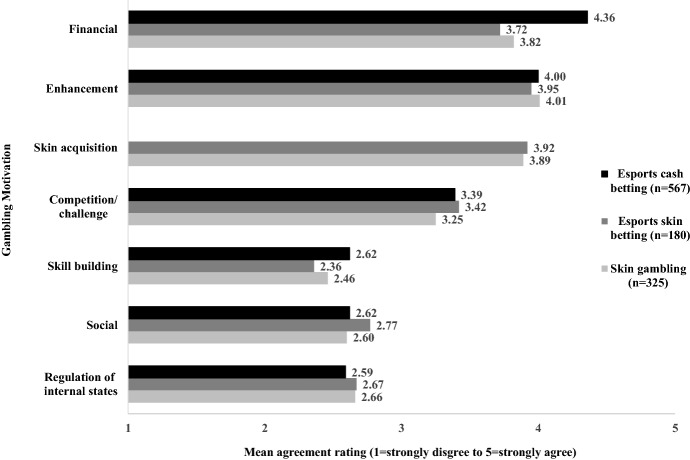
Mean gambling motivation scores for esports cash betting, esports skin betting, and skin gambling

Figure 1 shows the mean scores for each motivation domain for esports cash betting, esports skin betting, and skin gambling on games of chance. The primary motivation (i.e., the highest rated) for esports cash betting was the *financial* motive (“to win money”: M = 4.36, SD = 0.91), followed by *enhancement* (M = 4.00, SD = 0.88), and *competition/challenge* (M = 3.39, SD = 1.14). The strongest endorsed gambling motivation for esports skin betting was *enhancement* (M = 3.95; SD = 0.96), followed by *skin acquisition* (M = 3.92; SD = 0.90), *financial* (M = 3.72; SD = 1.12), and *competition/challenge* motives (M = 3.42; SD = 1.26). Motivations for skin gambling on games of chance showed similar patterns to esports skin betting, highly endorsing *enhancement* (M = 4.01; SD = 0.98), followed by *skin acquisition* (M = 3.89; SD = 0.99), *financial* (M = 3.82; SD = 1.06), and *competition/challenge* motives (M = 3.25; SD = 1.22). *Social*, *skill building*, and *regulation of internal states* for all three gambling activities were less strongly endorsed, with all mean scores for individual items and overall domain less than 3.00.

### Age and Gender Differences for Gambling Motivations

Correlational relationships between age and gambling motivations differed by gambling activity. For esports cash betting only the *competition/challenge* motivation was significantly correlated with age (r = 0.093, *p* = 0.026). Being older was correlated with higher scores on the *skill building* and *competition/challenge* motivations for esports skin betting (r = 0.248, *p* = 0.001 and r = 0.315, *p* < 0.001 respectively) and skin gambling (r = 0.224, *p* < 0.001 and r = 0.229, *p* < 0.001, respectively). *Regulation of internal states* (e.g., to escape from worries, to feel better) for esports skin betting was positively correlated with age (r = 0.155, *p* = 0.038). No age differences were found for the *social, financial, enhancement*, or *skin acquisition* motivations for any product.

Significant differences in gambling motivation scores were found by gender across the three products. For esports cash betting, females rated gambling motivations significantly higher than males for *social* (Females: M = 3.00, SD = 1.14; Males: M = 2.51, SD = 1.18; t = −4.101, *p* < 0.001), *skill building* (Females: M = 2.83, SD = 1.35; Males: M = 2.56, SD = 1.25; t = − 2.095, *p* = 0.037), *regulation of internal states* (Females: M = 2.87, SD = 1.32; Males: M = 2.51, SD = 1.26; t = − 2.73, *p* = 0.007), and *competition/challenge* (Females: M = 3.67, SD = 0.95; Males: M = 3.31, SD = 1.18; t = − 3.505, *p* = 0.001). In contrast, for esports skin betting, only *skill building* showed a gender difference, with females (M = 2.91, SD = 1.17) scoring higher than males (M = 2.27, SD = 1.22), t = − 2.357, *p* = 0.020. Lastly, for skin gambling on games of chance, females scored higher than males on *social* motivations (Females: M = 2.91, SD = 1.13; Males: M = 2.53, SD = 1.21; t = − 2.169, *p* = 0.031), *skill building* (Females: M = 3.25, SD = 1.24; Males: M = 2.30, SD = 1.23; t = − 5.291, *p* < 0.001), *regulation of internal states* (Females: M = 3.00, SD = 1.33; Males: M = 2.59, SD = 1.27; t = − 2.211, *p* = 0.028), and *competition/challenge* (Females: M = 3.68, SD = 1.08; Males: M = 3.16, SD = 1.23; t = − 2.928, *p* = 0.004). No gender differences were found for the *financial, enhancement*, or *skin acquisition* motivations for any product.

### Gambling Motivations as Predictors of Frequent Gambling

Table 5 shows the results of the three logistic regressions that examined gambling motivations as predictors of frequent gambling (at least monthly) on each of the three activities when controlling for age and gender. The *skill building* motivation was the only significant predictor for regular esports cash betting. *Regulation of internal states* and *competition/challenge* motivations significantly predicted regular esports skin betting. Frequent skin gambling on games of chance was predicted by being younger and motivated to gamble on that activity by *competition/challenge*.

**Table 5:** Logistic regressions predicting gambling frequency by gambling motivation (N = 736)

*Gambling motivation (IVs)*	*Gambling frequency (DVs)* (0 = less than monthly, 1 = at least monthly)											
	Esports cash betting (n = 567)				Esports skin betting (n = 180)				Skin gambling (n = 325)			
	Odds ratio	Confidence intervals		Cor	Odds ratio	Confidence intervals		Cor	Odds ratio	Confidence intervals		Cor
		Lower	Upper			Lower	Upper			Lower	Upper	
Age (years)	0.996	0.973	1.019	− .039	1.003	0.961	1.047	.134	0.964**	0.938	0.991	− .055
Gender (M, F)	0.663	0.430	1.022	− .064	0.829	0.304	2.265	− .006	0.759	0.391	1.473	− .013
Social	1.037	0.874	1.230	.063	0.884	0.648	1.205	.099	0.815	0.644	1.030	.035
Financial	1.156	0.950	1.405	.102*	1.141	0.837	1.555	.135	1.226	0.967	1.553	.169**
Enhancement	1.229	0.988	1.529	.109**	0.918	0.627	1.345	.123	0.958	0.741	1.240	.063
Skill building	1.241*	1.043	1.477	.160***	1.066	0.755	1.505	.208**	1.271	0.997	1.621	.166**
Regulation of internal states	1.088	0.920	1.288	.115**	1.408*	1.051	1.888	.271***	1.151	0.930	1.424	.151**
Competition/challenge	0.938	0.775	1.134	.081	1.547*	1.091	2.192	.294***	1.351*	1.064	1.717	.181**
Skin acquisition	–	–	–	–	0.940	0.640	1.380	.119	0.988	0.769	1.270	.062
Statistics	χ^2^ = 26.216, df = 8, *p* = .001, Pseudo R^2^ = 0.063				χ^2^ = 23.916, df = 9, *p* = .004, Pseudo R^2^ = 0.169				χ^2^ = 30.143, df = 9, p < .001, Pseudo R^2^ = 0.120			

*p < 0.05; **p < 0.01; ***p < 0.001; Cor = Spearman’s rho correlation; All measures rated on scale 1 = Strongly disagree to 5 = Strongly agree; Pseudo R^2^ = Nagelkerke. Financial motivation for esports cash betting contains single item “To win money”. Enhancement motivation for skin gambling contains single item “For the excitement”. Skin acquisition items not asked for esports cash betting

### Gambling Motivations as Predictors of Gambling Problems

Greater endorsement of the *regulation of internal states* motivation for all three activities significantly predicted being in a higher at-risk gambling category (PGSI), when controlling for age and gender (Table 6). The *financial* motivation for esports skin betting and skin gambling (but not esports cash betting) significantly predicted being in a higher at-risk gambling category (PGSI). Higher scores on the *skill building* motivation for esports cash betting predicted higher PGSI category, but not for esports skin betting or skin gambling.

**Table 6:** Ordinal regressions predicting problem gambling severity (PGSI) by gambling motivation (N = 736)

*Gambling motivation (IVs)*	*Problem Gambling Severity (DVs)* (PGSI 4 category)											
	Esports cash betting (n = 567)				Esports skin betting (n = 180)				Skin gambling (n = 325)			
	Odds Ratio	Confidence Intervals		Cor	Odds Ratio	Confidence Intervals		Cor	Odds Ratio	Confidence Intervals		Cor
		Lower	Upper			Lower	Upper			Lower	Upper	
Age (years)	0.964***	0.945	0.984	− .118**	0.951**	0.917	0.986	− .192*	0.956***	0.933	0.979	− .181**
Gender (M, F)	0.733	0.499	1.078	− .021	0.326*	0.139	0.765	− .162*	0.699	0.387	1.262	− .030
Social	1.146	0.986	1.331	.207***	1.113	0.855	1.450	.160*	1.124	0.914	1.381	.173**
Financial	1.100	0.921	1.313	.083*	1.441**	1.105	1.878	.285***	1.501***	1.217	1.851	.247***
Enhancement	0.920	0.756	1.119	.055	0.793	0.572	1.099	.088	0.819	0.652	1.028	.010
Skill building	1.250**	1.079	1.447	.306***	1.164	0.868	1.560	.204**	1.222	0.987	1.514	.233***
Regulation of internal states	1.831***	1.575	2.130	.425***	1.826***	1.398	2.385	.367***	1.758***	1.442	2.144	.368***
Competition/challenge	0.928	0.788	1.093	.159**	0.913	0.680	1.227	.089	0.842	0.680	1.043	.029
Skin acquisition	–	–	–	–	1.076	0.781	1.481	.113	0.951	0.760	1.190	.003
Statistics	χ^2^ = 141.372, df = 8, *p* = < .001, Pseudo R^2^ = 0.236				χ^2^ = 56.943, df = 9, *p* = < .001, Pseudo R^2^ = 0.290				χ^2^ = 89.154, df = 9, *p* = < .001, Pseudo R^2^ = 0.258			

*p < 0.05; **p < 0.01; ***p < 0.001; Cor = Spearman’s rho correlation; All motivations rated on scale 1 = Strongly disagree to 5 = Strongly agree; Pseudo R^2^ = Nagelkerke. Financial motivation for esports cash betting contains single item “To win money”. Enhancement motivation for skin gambling contains single item “For the excitement”. Skin acquisition items not asked for esports cash betting. PGSI = Problem Gambling Severity (0 = non-problem, 1 = low-risk, 2 = moderate-risk, 3 = problem)

### Gambling Motivations as Predictors of Gambling-Related Harm

Being *more* motivated by *regulation of internal states* (e.g., escape) for esports cash betting, esports skin betting, and skin gambling (games of chance) predicted greater gambling-related harm (Table 7). In addition, higher *financial* motivation scores for skin gambling predicted greater gambling-related harm, but not for esports cash betting or esports skin betting.

**Table 7:** Ordinal regressions predicting gambling harm (SGHS) by gambling motivation (N = 736)

*Gambling motivation (IVs)*	*Gambling-related harm (DVs)* (SGHS 4 category)											
	Esports cash betting (n = 567)				Esports skin betting (n = 180)				Skin gambling (n = 325)			
	Odds Ratio	Confidence Intervals		Cor	Odds Ratio	Confidence Intervals		Cor	Odds Ratio	Confidence Intervals		Cor
		Lower	Upper			Lower	Upper			Lower	Upper	
Age (years)	0.980*	0.961	1.000	− .076	0.969	0.936	1.003	− .157**	0.976*	0.953	0.999	− .112*
Gender (M, F)	0.847	0.576	1.247	− .010	0.746	0.317	1.760	− .065	0.852	0.478	1.521	− .010
Social	0.972	0.837	1.128	.103*	1.085	0.833	1.413	.017	0.955	0.784	1.164	.092
Financial	1.097	0.922	1.305	.105*	1.274	0.976	1.664	.196**	1.316**	1.071	1.618	.225***
Enhancement	1.101	0.904	1.340	.102*	1.090	0.790	1.503	.177*	1.057	0.843	1.326	.092
Skill building	1.077	0.930	1.246	.194***	0.973	0.720	1.315	.125	1.175	0.959	1.440	.164**
Regulation of internal states	1.650***	1.420	1.916	.335***	1.602***	1.231	2.084	.303***	1.414***	1.172	1.705	.258***
Competition/challenge	0.991	0.840	1.169	.137**	1.014	0.760	1.354	.114	0.911	0.743	1.115	.058
Skin acquisition	–	–	–	–	0.886	0.638	1.230	.053	1.046	0.833	1.314	.067
Statistics	χ^2^ = 77.596, df = 8, *p* = < .001, Pseudo R^2^ = 0.138				χ^2^ = 28.209, df = 9, *p* = .001, Pseudo R^2^ = 0.156				χ^2^ = 40.657, df = 9, p < .001, Pseudo R^2^ = 0.126			

*p < 0.05; **p < 0.01; ***p < 0.001; Cor = Spearman’s rho correlation; All motivations rated on scale 1 = Strongly disagree to 5 = Strongly agree; Pseudo R^2^ = Nagelkerke. Financial motivation for esports cash betting contains single item “To win money”. Enhancement motivation for skin gambling contains single item “For the excitement”. Skin acquisition items not asked for esports cash betting. SGHS = Short Gambling Harm Screen (0 = 0 harms, 1 = 1–2 harms, 2 = 3–4 harms, 3 = 5–10 harms)

## Discussion

This study examined motivations for esports cash betting, esports skin betting, and skin gambling on games of chance, whether these motivations differed by product, and associations between these motivations and gambling frequency, problems, and harm.

### Esports Cash Betting

Esports cash betting was primarily driven by the *financial* motivation “to win money” followed by *enhancement* motivations, with the latter being more so for excitement and less so for enhancing viewership of the esports match. These findings are consistent with quantitative research on motivations for traditional gambling activities (i.e., Flack & Morris, 2015; Francis et al., 2015; Lee et al., 2007; Stewart & Zack, 2008; Tabri et al., 2021) and qualitative research on sports betting motivations (Gordon et al., 2015; Lamont & Hing, 2018) that excitement and financial gain are primary gambling motives. The *competition/challenge* motivations were also moderately important to esports cash betting, with the “for the challenge” item rating slightly higher than “to compete with others”. The finding that challenge and competition are important aspects to esports cash betting is not surprising given the similarities between esports betting and traditional sports betting. For example, both products are offered by the same wagering operators, both involve competition (Gordon et al., 2015) and a high proportion of esports bettors also bet on traditional sports (Greer et al., 2021). Furthermore, while *skill building* was a less common motivation for esports cash betting, *skill building* was the only motivation that was positively associated with being both a regular esports cash bettor and higher problem gambling severity. It could be that esports cash betting offers an avenue to practice for similar gambling activities such as sports betting and in turn promotes more frequent gambling and the development of disordered gambling. In addition, erroneous cognitions that gambling outcomes are determined by skill rather than chance is considered a risk factor for problem gambling, and skill-building is a motivation supporting these cognitions (Abbott et al., 2018; Russell et al., 2019; Williams et al., 2012). Furthermore, being more motivated by *regulation of internal states* (escape, improve mood) for esports cash betting was significantly associated with higher problem gambling severity and experiencing more gambling-related harms. These results are consistent with similar findings on traditional gambling forms (Browne et al., 2019; Flack & Morris, 2015; Francis et al., 2015; Lambe et al., 2015; Rockloff & Dyer, 2006; Rodrigeuz et al., 2015; Stewart & Zack, 2008; Wardle et al., 2011). In addition, the findings align to theory that emotionally vulnerable gamblers and negative coping styles are associated with problem gambling (Abbott et al., 2018; Blaszczynski & Nower, 2002; Kurilla, 2021).

### Esports Skin Betting

In contrast, esports skin betting was highly driven by motivations around *skin acquisition* (i.e., collection, exchange for skins, use in video games), followed by *enhancement* (“for the excitement”) and the two *financial* motives (“to win skins to exchange to money”, then “to win money”). The *competition/challenge* motivations were similar to esports cash betting, being rated as moderately important. Additionally, higher *competition/challenge* motivations were associated with more frequent gambling for esports skin betting but not esports cash betting. The *competition/challenge* motives for esports skin betting may be more about obtaining skins than the esports competition, whether as a challenge for themselves (i.e., get a rare skin for their collection) or in competition with other video gamers. In turn, this competition may drive greater gambling frequency in the effort to obtain skins. However, *competition/challenge* motivations were not similarly predictive of downstream gambling problems or harm. Being more highly motivated for esports skin betting by *regulation of internal states* (escape, improve mood) and *financial* gain, in contrast, was associated with more frequent esports skin betting, greater problem gambling severity and harm – consistent with recent research with esports bettors (Lelonek-Kuleta & Bartczuk, 2021) and decades of gambling research investigating other forms (e.g., EGMs).

### Skins Gambling on Games of Chance

The main motivations for skin gambling on games of chance closely aligned with those for esports skin betting: *skin acquisition, enhancement,* and *financial* motives. The finding that winning skins for non-monetary gain was just as important as financial gain highlights the attraction of monetised video gaming activities where randomised items can be won, as observed in studies of loot boxes (Rockloff et al., 2020; Zendle et al., 2019). The finding that skin gambling was less motivated by *competition/challenge* than esports cash and skin betting reflects that, as games of chance, most skin gambling activities are played alone with randomly generated outcomes. Stronger *competition/challenge* motivations for skin gambling were associated with more frequent gambling on this activity, but again not problem or harmful gambling. As before, higher motivations for *regulation of internal states* for skin gambling on games of chance was associated with greater problem gambling severity, and gambling-related harms. Lastly, *financial* motivations for skin gambling were associated with both greater problem gambling severity and gambling-related harms. These findings position skin gambling as comparable to monetary forms of gambling, which when motivated by financial gain also increase the likelihood of harmful gambling (Browne et al., 2019; Dechant, 2014; Francis et al., 2015; Lee et al., 2007; Tabri et al., 2021). It is notable that skin gambling on games of chance is functionally equivalent to other forms of gambling (e.g., EGMs, casino games), with the exception that the underlying currency is skins rather than fiat currency.

### Limitations and Future Research

There are some limitations of this research which should be considered. First, the findings are only generalisable to the sampled adults who were predominately young adult males. This aligns with research indicating that esports bettors are more likely to be young males (i.e., Browne et al., 2019; Gainsbury et al., 2017a). Nonetheless, recruiting participants via social media and crowdsourcing, as well as non-completions and those who failed data quality checks, may have biased to the final sample characteristics. Further research is needed on the extent and impact of motivations for gambling on emerging products in the general adult population, as well as amongst children and adolescents. Second, the cross-sectional nature of the research only infers associations between variables and not necessarily causality, which would be better captured by longitudinal research. Third, in the logistic regression analyses the odds ratios for gambling motivations which significantly predicted regular gambling, problem gambling, and gambling-related harm were all relatively small and the total variance of the dependent variables explained ranged between 6 and 29%. This suggests other factors in addition to age, gender, and gambling motivations influence gambling frequency, problematic gambling, and gambling-related harm. Future research could examine gambling motivations for these products while accounting for other variables known to contribute to harmful gambling, such as gambling behaviours (frequency, money, time), impulsivity, and erroneous gambling cognitions (Browne et al., 2019; Lambe et al., 2015; Russell et al., 2019). Lastly, the current study assessed gambling motivations based on validated measures of traditional gambling motivations, online gaming motivations, and qualitative research. Future studies should look to validate measures of gambling motivations independently for esports cash betting, esports skin betting, and skin gambling.

## Conclusions

The current study provides insights into why people gamble on esports and with skins, which has been a relatively unexplored area of research. Engagement in esports cash betting, esports skin betting, and skin gambling on games of chance all appear to be motivated primarily by enhancing positive feelings such as excitement, winning money directly or via exchanging skins; i.e., for largely non-monetary purposes. The importance of skin acquisition is currently unique to esports skin betting and skin gambling, distinguishing it from esports cash betting and other traditional monetary forms of gambling. Despite the importance of skin acquisition, there was no evidence that this motivation was associated with more regular, problematic, or harmful gambling. However, motivations of financial gain via esports skin betting and skin gambling (games of chance) were associated with greater problem gambling severity. In line with traditional gambling activities, negative reinforcement, i.e., esports betting or skin gambling to relieve negative emotions or to escape, was associated with being at greater risk for experiencing gambling problems and harm. Further research is needed to confirm these results. Replication of these findings in representative samples would suggest that education and public health programs should attempt to dissuade young people from engaging in esports betting and skin gambling as a means of financial gain or as a way to escape from negative emotions (Rockloff et al., 2011).

## Data Availability

Data and material not currently available.
